# The Interaction of Lean Body Mass With Fat Body Mass Is Associated With Vertebral Fracture Prevalence in Women With Early Breast Cancer Undergoing Aromatase Inhibitor Therapy

**DOI:** 10.1002/jbm4.10440

**Published:** 2020-12-21

**Authors:** Sara Monteverdi, Rebecca Pedersini, Fabio Gallo, Filippo Maffezzoni, Alberto Dalla Volta, Pierluigi Di Mauro, Antonella Turla, Lucia Vassalli, Mara Ardine, Anna Maria Formenti, Edda Lucia Simoncini, Andrea Giustina, Roberto Maroldi, Vito Amoroso, Alfredo Berruti

**Affiliations:** ^1^ Department of Medical and Surgical Specialties, Radiological Sciences and Public Health, Medical Oncology University of Brescia, Aziende Socio Sanitarie Territoriali (ASST) Spedali Civili Brescia Italy; ^2^ Breast Unit ASST‐Spedali Civili Brescia Italy; ^3^ Clinical Epidemiology Unit, IRCCS, Ospedale Policlinico San Martino Genoa Italy; ^4^ Department of Medical and Surgical Specialties, Radiological Sciences and Public Health, Radiology University of Brescia, Aziende Socio Sanitarie Territoriali (ASST) Spedali Civili Brescia Italy; ^5^ Department of Endocrinology, San Raffaele Vita‐Salute University and Division of Endocrinology San Raffaele Istituto di Ricovero e Cura a Carattere Scientifico (IRCCS) Hospital Milan Italy

**Keywords:** BODY COMPOSITION, VERTEBRAL FRACTURES, AROMATASE INHIBITORS, BREAST CANCER

## Abstract

Aromatase inhibitors (AIs) induce depletion of estrogen levels, causing bone loss and increased fracture risk in women with breast cancer. High‐fat body mass (FBM) emerged as an independent factor associated with the prevalence of morphometric vertebral fractures (VFs) in patients undergoing AIs. We explored the role of lean body mass (LBM) and the interaction of LBM with FBM in predicting the occurrence of VFs in postmenopausal women who were either AI‐naïve or AI‐treated. A total of 684 consecutive breast cancer patients were enrolled in this cross‐sectional study. Each woman underwent a dual‐energy X‐ray absorptiometry (DXA) scan, measuring bone mineral density (BMD), LBM, and FBM; VFs were assessed using a quantitative morphometric analysis of DXA images. After propensity score matching, the study population was restricted to 480 women, 240 AI‐naïve and 240 AI‐treated. We used multivariable logistic regression models to explore the associations between baseline characteristics, VF prevalence and the interaction between LBM, FBM and AI therapy. No interaction between LBM and AI therapy on VF prevalence was shown. Conversely, we reported a significant interaction between LBM, FBM and AI therapy (*p* = .0311). Among AI‐treated women having LBM below and FBM above or equal the median value, VF prevalence was numerically higher (15/31; 48.4%) than in other subgroups (VF prevalence: 35.7% in high‐LBM and low‐FBM group, 23.2% in high‐LBM and high‐FBM group, and 19.8% in low‐LBM and low‐FBM group). Among AI‐naïve women, the greatest VF proportion was observed in the subgroup with LBM and FBM below median value (25/92; 27.2%). This study suggests a synergism between LBM and FBM in predicting the morphometric VF in women with early breast cancer undergoing AIs. This observation is new and deserves further investigation. The assessment of body composition by DXA might be useful when estimating fracture risk in this population. © 2020 American Society for Bone and Mineral Research © 2020 The Authors. *JBMR Plus* published by Wiley Periodicals LLC. on behalf of American Society for Bone and Mineral Research.

## Introduction

Aromatase inhibitors (AIs) are widely used as adjuvant therapy for postmenopausal women with endocrine‐sensitive early breast cancer (BC). These drugs, although effective in prolonging survival, may cause bone fragility.^(^ ^1^ ^)^ Primary prevention of AI‐mediated fractures has become a clinical priority because their incidence can reach 20% after 5 years of follow‐up, with a further increase of 2% to 3% per year for treatments up to 10 years.^(^ ^2^ ^)^

AIs cause a more rapid and severe form of skeletal fragility as compared to postmenopausal osteoporosis,^(^ ^3^ ^)^ which cannot be reliably predicted by bone mineral density (BMD) measurement^(^ ^4^ ^)^ because it is mainly caused by an early impairment of bone quality (ie, trabecular microarchitecture). This became increasingly evident when vertebral fractures (VFs), historically underestimated and often clinically undiagnosed, were proactively searched with the modern morphometric approach.^(^ ^5^, ^6^ ^)^ As a matter of fact, a randomized clinical trial has revealed that in postmenopausal women with early BC on adjuvant AI therapy, there was no difference in morphometric VF risk among patients with low versus normal BMD.^(^ ^7^ ^)^

New predictors of fracture risk in AI‐treated patients are needed to tailor the best preventive measures for each patient. In our previous study, postmenopausal women receiving AI therapy that had high‐fat body mass (FBM), as measured by dual‐energy X‐ray absorptiometry (DXA), had an increased prevalence of morphometric VFs as compared with women with low‐FBM.^(^ ^8^ ^)^ Detrimental effects of adiposity on bone quality could be hypothesized through altered bone‐regulating hormones, including vitamin D,^(^ ^9^ ^)^ increased oxidative stress, and inflammation, combined with bone effects induced by the low levels of estrogens with AI therapy.^(^ ^8^, ^10^, ^11^ ^)^

However, the increasing FBM could not be considered per se the only risk factor of VF, because also the concomitant decrease of lean body mass (LBM) may be hypothesized to contribute to skeletal fragility in this setting. In fact, in recent years, the concept of a bone‐muscle unit has emerged, based on space flight and immobilization, as well as osteoporosis and sarcopenia studies.^(^ ^12^ ^)^ Mechanical loading is a key mechanism linking both muscle and bone, with a central promoting role of physical activity, which may favor muscle degradation and bone resorption if decreased. Moreover, the skeletal muscle may produce several substances that can positively influence bone metabolism, including but not limited to insulin‐like growth factor‐1 (IGF‐1),^(^ ^13^, ^14^ ^)^ myostatin, basic fibroblast growth factor, and interleukin‐6 (IL‐6). Cartilage and adipose tissue are also likely to participate in these control mechanisms.^(^ ^12^ ^)^

Sarcopenia is an independent risk factor for fragility fractures in healthy postmenopausal women, leading to a condition known as sarco‐osteoporosis.^(^ ^15^, ^16^, ^17^ ^)^ The negative effect of sarcopenia can increase if obesity coexists. Moreover, in the elderly population, sarcopenia and obesity have demonstrated independent and cumulative adverse effects on bone health.^(^ ^18^ ^)^ Finally, sarcopenia occurs in over one‐third of newly diagnosed patients with nonmetastatic BC and has a relevant prognostic impact.^(^ ^19^ ^)^

These pieces of knowledge provide the rationale to explore the effects of LBM and the interaction between LBM and FBM, as assessed by DXA, on the prevalence of morphometric VFs in postmenopausal women on AI therapy. We aimed to identify factors potentially predicting the fracture risk in this population beyond BMD. For this purpose, we used a prospectively collected dataset from a study approved and conducted at a single institution, whose preliminary results were previously reported.^(^ ^4^, ^8^ ^)^

## Patients and Methods

### Study design and patient population

This cross‐sectional study followed the Strengthening the Reporting of Observational Studies in Epidemiology reporting guideline.^(^ ^20^ ^)^ The study protocol and patient population were previously described in detail.^(^ ^4^, ^8^ ^)^ Briefly, eligible patients were postmenopausal women with hormone receptor‐positive BC (pathologic stage I‐III according to American Joint Committee on Cancer staging system), who were candidates to adjuvant endocrine therapy with an AI, and without any bone metabolic diseases, with normal renal function and no previous or current treatment with anti‐osteoporotic drugs or glucocorticoids. The study cohort was enlarged with respect to the last report,^(^ ^8^ ^)^ and the updated database was locked on November 2, 2019. The dataset used for the present analyses included 684 cases: 439 patients assessed before initiating adjuvant endocrine therapy (AI‐naive group) and 245 patients assessed while receiving adjuvant AI therapy for at least 2 years (AI‐treated group).

### Assessments

Each patient underwent a DXA scan (Explorer; Hologic, Inc., Marlborough, MA, USA), assessing BMD at the vertebral, hip, and femoral level, FBM and LBM,^(^ ^21^ ^)^ and VFs. DXA analyses were performed by two experienced physicians (FM and AMF) who underwent a specific training course for this study. Before starting the study enrollment, 30 patients were evaluated blindly by both physicians to assess coefficients of variation for FBM and LBM (1.5%). VFs were assessed by the two physicians, who were blinded to patient group assignment, using a quantitative morphometric analysis of DXA images. Using a translucent digitizer and a cursor, six points were marked on each vertebral body to describe the vertebral shape. Anterior (Ha), middle (Hm), and posterior (Hp) vertebral heights were measured, and height ratios (Ha/Hp, Hm/Hp, Hp/Hp of the above vertebrae, Hp/Hp of the below vertebrae) were calculated for each vertebra body from T_5_ to L_4_; the VFs were classified as mild (height ratio decrease of 20% to 25%), moderate (decrease of 26% to 40%), or severe (decrease of >41%).^(^ ^22^ ^)^ The intraobserver and interobserver coefficients of variation were between 1% and 4% and 3% to 6%, in relation to the site (lumbar versus thoracic) and severity (mild versus severe) of fractures. Discordant cases were solved by consensus.

### Statistical analysis

Continuous variables were given as means with standard deviations (SDs) and categorical variables as number of subjects and percentage values. To obtain a more similar set of participants in each group, a propensity score nearest neighbor matching was performed. Baseline characteristics used to estimate the propensity score were age, previous fractures, chemotherapy use, alcohol consumption, physical activity, and body mass index (BMI). The balanced post‐match analysis was performed using univariate logistic regression models, as well as graphical approaches.

The body composition features based on the LMB and FBM distribution were identified as follows: group A, patients with both LBM and FBM below the median value; group B, patients with LBM below the median value and FBM above or equal the median value; group C, patients with LBM above or equal the median value and FBM below the median value; group D, patients with both LBM and FBM above or equal the median value. The cutoff of LBM and FBM median value was arbitrarily predefined to explore the possible interaction between body composition parameters and VF prevalence.

After that, univariate logistic regression models were performed to screen the effect of the clinical and demographic variables on the prevalence of morphometric VFs. Those covariates with a *p* value <.05 were then selected for the multivariable analysis, where the VF prevalence was the dependent variable. The multivariable analysis was performed using again the logistic regression model. The odds ratios (ORs) associated with the VF prevalence were calculated with their 95% confidence interval (CI) for each factor from the logistic regression model. The likelihood ratio test was used as a test of statistical significance, and the model selection was performed using the Akaike's Information Criterion.

Moreover, multiplicative interaction terms were used to test whether the LBM and the body composition effects on the VFs were different according to the AI group. For those results suggestive of an interaction (*p* value <.05), a stratified analysis was then performed based on AI group using the penalized logistic regression model. Because of the explorative nature of this study, formal sample size was not calculated.

Finally, using the log‐transformation of the LBM and FBM continuous variables, a multivariate model with multiplicative interaction terms was performed to test whether the LBM and FBM effects on the VFs were different according to the AI group. The predicted probability plot of VF based on LBM and FBM in the AI‐naïve and AI‐treated group was drawn for those results suggestive of an interaction (*p* value <.05).

The estimated *p* values were adjusted for multiple comparisons by the Holm correction method. Differences with a *p* value <.05, were selected as significant, and data were acquired and analyzed in the R v3.6.3 software environment (R Foundation for Statistical Computing, Vienna, Austria; https://www.r-project.org/).^(^ ^23^ ^)^

## Results

A total of 684 subjects participated in this study. The demographic and clinical characteristics of the study participants are summarized in Table 1. Patients characteristics before and after propensity score matching are shown in Table S1.

**Table 1:** Demographic and Clinical Characteristics of Study Participants (*n* = 684)

Characteristic	Overall
Morphometric vertebral fracture, *n* (%)	
Absence	551 (80.6)
Presence	133 (19.4)
Vertebral fracture grade, *n* (%)	
No	551 (80.6)
Mild	66 (9.6)
Moderate/severe	67 (9.8)
AI group, *n* (%)	
AI‐naïve	439 (64.2)
AI‐treated	245 (35.8)
Age (years)	63.0 ± 9.9
BMI (kg/m^2^)	25.7 ± 4.6
Physical activity, *n* (%)	
No	454 (73.1)
Yes	167 (26.9)
Smoking status, *n* (%)	
No	559 (84.3)
Yes	104 (15.9)
Alcohol consumption, *n* (%)	
No	500 (78.1)
Yes	140 (21.9)
Pathologic tumor stage, *n* (%)	
pT1	467 (69.0)
pT2	186 (27.5)
pT3‐4	24 (3.5)
Pathologic nodal status, *n* (%)	
pN0	405 (60.4)
pN1	225 (33.5)
pN2‐3	41 (6.1)
Chemotherapy, *n* (%)	
No	422 (61.9)
Yes	260 (38.1)
Previous fractures, *n* (%)	
No	543 (81.9)
Yes	120 (18.1)
Lumbar spine BMD (g/cm^2^), mean ± SD	0.89 ± 0.14
Lumbar spine *T*‐score, mean ± SD	−1.33 ± 1.36
Femoral neck BMD (g/cm^2^), mean ± SD	0.69 ± 0.1
Femoral neck *T*‐score, mean ± SD	−1.43 ± 0.91
Total hip BMD (g/cm^2^), mean ± SD	0.82 ± 0.11
Total hip *T*‐score, mean ± SD	−1.01 ± 0.89
Lean body mass (g), mean ± SD	39,977.34 ± 5062.1
Fat body mass (g), mean ± SD	26,163.43 ± 23,634.3

The results are expressed as mean ± SD or as number of subjects with percentage.

AI = aromatase inhibitor; BMD = bone mineral density; BMI = body mass index.

In the complete dataset, the prevalence of morphometric VFs was significantly higher in AI‐treated than AI‐naïve patients, 64 out of 245 (26%) versus 69 out of 439 (16%), respectively (OR 1.90; 95% CI, 1.29–2.78; *p* = .020) and patients were older in AI‐treated as compared with AI‐naïve group (mean ± SD age 66.3 ± 7.7 versus 61.2 ± 10.5 years, *p* < .001). The proportion of moderate or severe VFs was 13% in AI‐treated patients and 8% in AI‐naïve ones (*p* = .074). No significant difference in mean LBM and FBM values was observed in AI‐treated versus AI naïve patients.

Among the 684 women, 480 could be matched with a propensity score, 240 in each group, AI‐naive and AI treated. The matched groups were found to be well‐balanced without significant differences among baseline characteristics (Table S1).

### Factors associated with the VF prevalence

Descriptive statistics of the demographic and clinical characteristics of women with the presence of VFs versus those with the absence of VFs after propensity score adjustment are reported in Table S2. Briefly, the mean age of patients without VFs was 65.1 ± 7.6 years, whereas the mean age of patients with VFs was 69.5 ± 7.2 years (*p* < .001). The presence of previous fractures was higher in patients with prevalent VFs than in patients without VFs, 42% versus 17%, respectively (*p* < .001).

The univariate logistic regression analysis, using the complete set of data after propensity score adjustment (Table S2), demonstrated a significant association between age, previous fractures, total hip BMD, total hip *T*‐score and the occurrence of VF (*p* values: <.001, <.001, .016, and .015, respectively).

### The role of LBM and the interaction LBM/FBM in predicting the prevalence of VF

In the propensity score‐matched sample, the body composition groups based on LMB and FBM categorized at the median value were distributed as follows: group A (178 patients; 37%); group B (62 patients; 13%); group C (61 patients; 13%); and group D (179 patients; 37%).

Significant univariate characteristics and the multiplicative interaction terms entered in the multivariable logistic regression analysis. In particular, the LBM effect on the VF prevalence was not significantly different according to the AI groups (*p* value for interaction = .596). Conversely, a statistically significant effect of age, previous fractures, and the interaction term of LBM, FBM, and AI group upon the VF prevalence was observed (Table 2; *p* values: <.001, <.001, and .003, respectively). Specifically, a 1‐year increase in age was associated with an increased 9% chance of a prevalent VF, maintaining constant the other covariates (OR 1.09; 95% CI, 1.05–1.13). Taking as reference patients without previous fractures, the chance of a prevalent VF was about 4.5 times more likely in patients with previous fractures, keeping constant the other covariates (OR 4.47; 95% CI, 2.65–7.63).

**Table 2:** Multivariable Logistic Regression Analysis in the Propensity Score‐Matched Sample (*n* = 480): The Predictor Effects on the Prevalence of Morphometric Vertebral Fractures

	Multivariable analysis	
Characteristic	OR (95% CI)	*p* ^a^
Age	1.09 (1.05–1.13)	<.001
Previous fractures		<.001
No	1	
Yes	4.47 (2.65–7.63)	
Total hip BMD, (g/cm^2^)	0.18 (0.02–2.11)	.17
AI group		.18
AI‐naïve	1	
AI‐treated	0.59 (0.27–1.26)	
Body composition		.43
Group A: LBM− and FBM−	1	
Group B: LBM− and FBM+	0.45 (0.13–1.32)	
Group C: LBM+ and FBM−	0.42 (0.12–1.26)	
Group D: LBM+ and FBM+	0.45 (0.19–1.05)	
AI group * body composition		.003
AI‐naïve/Group A	1	
AI‐treated/Group B	11.72 (2.68–57.29)	
AI‐treated/Group C	6.79 (1.52–33.14)	
AI‐treated/Group D	2.2 (0.71–6.98)	

Results are expressed as odds ratio (OR) with 95% confidence interval (95% CI).

AI = aromatase inhibitor; BMD = bone mineral density; FBM− = fat body mass < median value; FBM+ = fat body mass ≥ median value; LMB− = lean body mass < median value; LBM+ = lean body mass ≥ median value.

^a^ Likelihood ratio *p* value adjusted for multiple comparisons.

Concerning the multiplicative interaction term, the effect of the body composition variable (based on LBM and FBM dichotomized at the median values) on the prevalence of VFs was significantly different according to the AI group (*p* value for the interaction term = .003). The subsequent stratification analysis (Table 3) showed that the condition of LBM below the median and FBM above or equal the median (group B) was significantly associated with about a 4.8‐fold increased chance of having a prevalent VF in AI‐treated patients (OR 4.81; 95% CI, 1.82–13.21) as compared with the reference group (group A). In the stratified analysis, we also observed a 2.7 increase in VF chances for group C (high LBM and low FBM) in the AI‐treated women, but this association was not statistically significant (OR 2.68; 95% CI, 0.97–7.36). The forest plot of this stratification analysis is reported in Fig. S3.

**Table 3:** Stratification Analysis by AI Group on the Chance of a Prevalent Vertebral Fracture With Covariate Adjustment After Propensity Score Matching

	AI‐naïve group (*n* = 240)			AI‐treated group (*n* = 240)		
	Descriptive statistics: Morphometric VF			Descriptive statistics: Morphometric VF		
Characteristic	Absence (*n* = 193) (80.4%)	Presence (*n* =47) (19.6%)	OR (95% CI)	Absence (*n* = 176) (73.3%)	Presence (*n* = 64) (26.7%)	OR (95% CI)
Age (years), mean ± SD	64.9 ± 7.9	69.5 ± 7.4	1.08 (1.03–1.13)	65.4 ± 7.3	69.5 ± 7.2	1.09 (1.04–1.15)
Previous fractures, *n* (%)						
No	162 (84.4)	30 (15.6)	1	144 (80.9)	34 (19.1)	1
Yes	31 (64.6)	17 (35.4)	3.2 (1.47–6.98)	32 (51.6)	30 (48.4)	5.26 (2.63–10.87)
Total hip BMD (g/cm^2^), mean ± SD	0.84 ± 0.11	0.76 ± 0.1	0.01 (0–0.32)	0.81 ± 0.09	0.81 ± 0.12	3.02 (0.11–91.37)
Body composition, *n* (%)						
Group A, LBM− and FBM−	67 (72.8)	25 (27.2)	1	69 (80.2)	17 (19.8)	1
Group B, LBM− and FBM+	26 (83.9)	5 (16.1)	0.54 (0.16–1.56)	16 (51.6)	15 (48.4)	4.81 (1.82–13.21)
Group C, LBM+ and FBM−	28 (84.8)	5 (15.2)	0.63 (0.19–1.89)	18 (64.3)	10 (35.7)	2.68 (0.97–7.36)
Group D, LBM+ and FBM+	72 (85.7)	12 (14.3)	0.62 (0.26–1.45)	73 (76.8)	22 (23.2)	0.83 (0.37–1.88)

AI = aromatase inhibitor; BMD = bone mineral density; FBM− = fat body mass < median value; FBM+ = fat body mass ≥ median value; LBM+ = lean body mass ≥ median value; LMB− = lean body mass < median value; VF = vertebral fractures.

**Fig 1:**
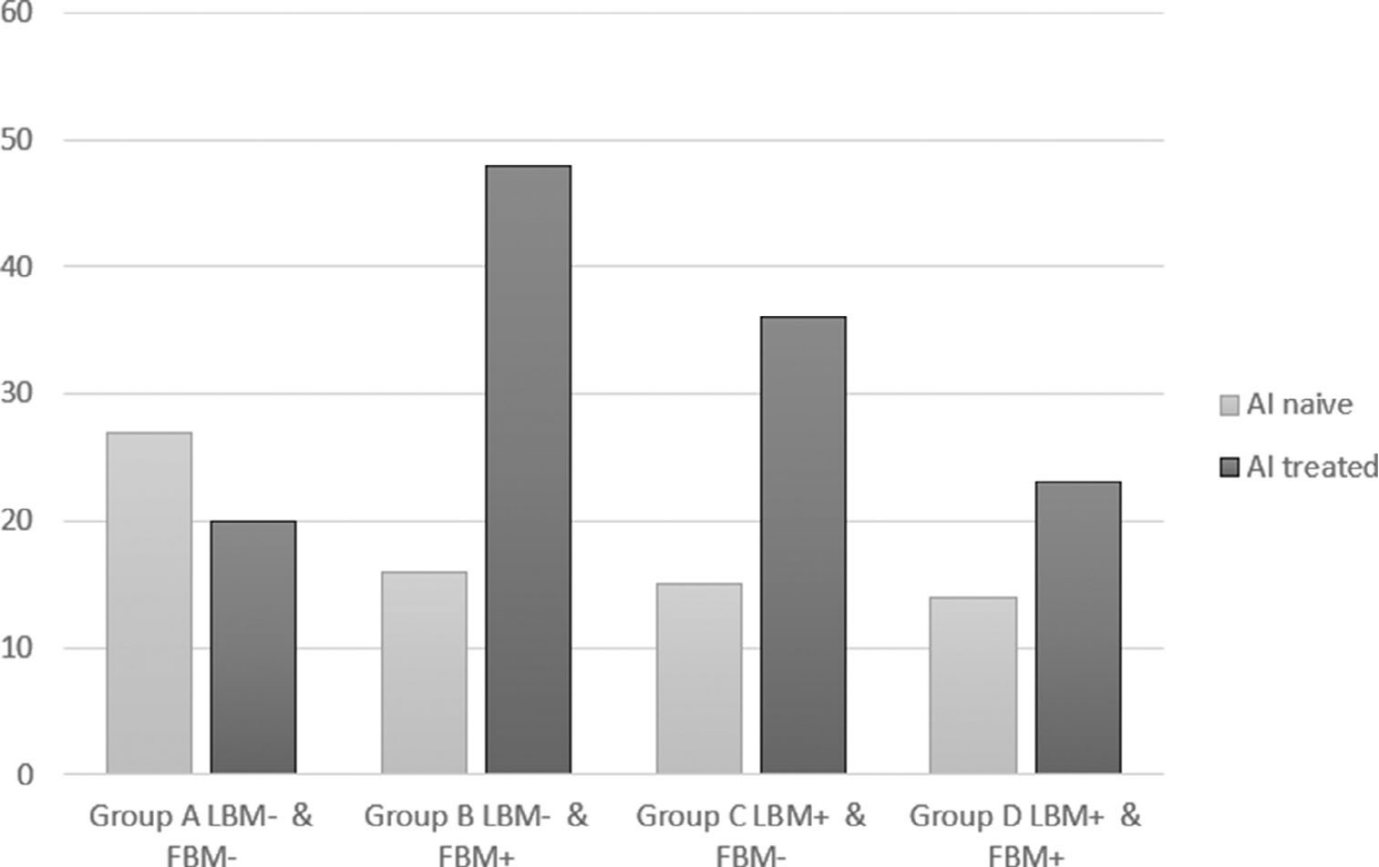
Vertebral fracture prevalence stratified according to body composition in AI‐naïve and AI‐treated patients. AI = aromatase inhibitor; LMB− = lean body mass < median value; LBM+ = lean body mass ≥ median value; FBM− = fat body mass < median value; FBM+ = fat body mass ≥ median value.

As shown in Fig. 1, among AI‐naive patients, the greatest proportion of VFs was observed in group A, with both low‐LBM and FBM (25/92; 27.2%), whereas in the AI‐treated women, the numerically highest prevalence of VFs was reported in group B, with low‐LBM and high‐FBM (15/31; 48.4%) as compared with other groups (VF prevalence: 35.7% in high‐LBM and low‐FBM group; 23.2% in high‐LBM and high‐FBM group; and 19.8% in low‐LBM and low‐FBM group).

In the multivariable model with LBM and FBM treated as continuous variables, the effect of the LBM and FBM on the prevalence of VFs was also significantly different according to the AI group (Table S4; *p* value for the interaction term = .0311). The predicted probability plot of VF based on LBM and FBM in the AI‐naïve and AI‐treated groups was reported in Fig. S5. In detail, fixing the FBM at the minimum value, we observed a slight decrease of the predicted probability of VF with increasing LBM values in the AI‐naïve group (red line, Fig. S5A) and a sharp increase of the predicted probability of VF with high‐LBM values in the AI‐treated group (red line, Fig. S5B). Conversely, when we fixed the FMB at the maximum value, we observed a slight increase of the predicted probability of VF with increasing LBM values in the AI‐naïve group (green line, Fig. S5A) and a progressive and constant decrease of the predicted probability of VF with increasing LBM values in the AI‐treated group (green line, Fig. S5B).

## Discussion

LBM, as measured by DXA, was not associated with the VF prevalence in this cross‐sectional study, either in the overall study population of early BC women or stratifying patients according to whether they were AI‐naïve or AI‐treated. Moreover, we explored the potential interaction between LBM and FBM in predicting the VF, a known marker of frailty and poor prognosis, as well as a clinical parameter associated with reduced quality of life.^(^ ^24^ ^)^ Our analyses showed that this interaction is statistically significant in the AI‐treated subset, but not in the AI‐naïve group. The present single‐center study included 684 early BC patients; 556 of these women were already analyzed in a previous report.^(^ ^8^ ^)^ We reported that the phenotype characterized by low‐LBM and high‐FBM is not rare (13% of cases), although the cutoff at the median value we adopted could not fit with the definition of sarcopenic obesity described in the literature.

To examine in more depth our results and give some starting points for further studies, we also performed a multivariable model with a multiplicative interaction term using the LBM and FBM as continuous variables. In this model, the third‐degree interaction with two continuous and one categorical variable (AI group) was statistically significant (*p* = .0311). We showed that the VF predicted probability steadily decreased with increasing LBM in the AI‐treated group when we fixed the FBM at the maximum value. Conversely, and unexpectedly, the VF probability increased with high‐LBM values in the AI‐treated group when we set the FBM at the minimum value. In the AI‐naïve group, we did not observe any particular trend of the VF predicted probability with increasing LBM after fixing the FBM at the minimum or maximum value.

These data are consistent with the results of a large study by the Women's Health Initiative (WHI), in which women with sarcopenia alone were at similar risk of hip fracture than non‐sarcopenic women.^(^ ^25^ ^)^ To the best of our knowledge, this is the first study examining whether lean mass or the interaction of lean mass with fat mass can predict the occurrence of VFs in women with early BC.

There is growing interest in the crosstalk between muscle and bone. Several studies have reported a strong positive association between low muscle mass and poorer bone quality, mostly due to lower mechanical load.^(^ ^26^ ^)^ Moreover, sarcopenia is frequently found and has negative prognostic significance in women with BC, and sarcopenic obesity is a phenotype emerging in the general population, although still not completely defined.^(^ ^27^ ^)^

Fat mass notoriously has a protective role on the fracture risk in healthy women and men through an increased BMD mediated by higher estrogen levels due to greater aromatase activity. This protective effect is lost in women undergoing AIs, leading to adiposity's adverse effects on the bone to prevail. In a smaller cohort of patients, also included in the present analysis, we recently observed an independent direct relationship between high‐FMB and morphometric VF prevalence in AI‐treated patients. In contrast, an opposite association was noted in the AI‐naive ones.^(^ ^8^ ^)^

The coexistence of reduced muscle mass and increased adiposity was shown to be associated with a worse prognosis in early BC.^(^ ^19^ ^)^ Moreover, osteosarcopenic obesity was associated with frailty and poor physical performance, in terms of muscle strength and function, in middle‐aged and older women.^(^ ^28^ ^)^

As expected, among AI‐naïve patients, we observed the highest proportion of fractures in the pure catabolic subgroup with low‐LBM and low‐FBM. Conversely, in the AI‐treated subset, the numerically highest fracture rate was observed in women with the coexistence of low‐LBM and high‐FBM, although VF prevalence was unexpectedly high also in the subgroup with high‐LBM and low‐FBM.

These data provide additional information compared to our previous study.^(^ ^8^ ^)^ From the current results, it seems that it is not the FBM itself but rather the interaction between FBM and LBM that favors the onset of bone fractures in women who undergo treatment with AIs.

Due to the already well‐described interplay between bone, muscle, and adipose tissue,^(^ ^29^ ^)^ obesity and sarcopenia may be synergistic in favoring bone fragility and disability, leading to the concept of osteosarcopenic obesity as an evolution of the term sarcopenic obesity.^(^ ^27^ ^)^ However, to prove the clinical relevance of this new phenotype, an impact on the significant endpoint of bone metabolism, ie, fractures, is needed, and yet to be demonstrated.^(^ ^30^ ^)^ The higher estrogen levels in obese people and the positive effects of estrogens on BMD may mask the synergistic effect between obesity and sarcopenia on bone fragility in postmenopausal women. This is not the case in BC patients under AI therapy, and the results of the present study show that the interaction between low‐LBM and high‐FBM in favoring bone fractures becomes clearly evident in a condition of estrogen deprivation.

Our study further suggests that the pathophysiology underlying bone fragility may be different in women undergoing AIs than women with postmenopausal osteoporosis. Another interesting finding of our research is that BMD is not reliable in predicting VFs in AI‐treated women, as described.^(^ ^4^ ^)^ Inasmuch, identification of the body composition phenotype associated with a higher risk of fracture may help in the management of the bone outcomes of BC patients treated with AIs. In fact, at odds with recommendations of international guidelines,^(^ ^31^ ^)^ the use of BMD and the FRAX score (from the Fracture Risk Assessment Tool), which identifies low BMI as a risk factor of fractures, could only detect a minority of women with BC at higher risk of fracture. We suggest instead that the proactive identification of the unfavorable body composition phenotype (with high‐FBM and low‐LBM) may significantly add to an effective bone health management of women undergoing AI therapy. Furthermore, selective nutritional approaches and physical activity programs could be hypothesized to be helpful in fracture prevention programs in this clinical setting.^(^ ^32^ ^)^

The patient stratification according to the median values of LBM and FBM showed a tendency toward an increase in fractures also in AI‐treated patients with high‐LBM and low‐FBM as compared with their counterparts with low‐LBM and low‐FBM. This finding, although not significant, is unexpected and needs confirmation. However, an AI‐mediated unbalance between circulating estrogens and androgens has been described^(^ ^33^ ^)^ and might be hypothesized to be responsible for this body composition phenotype.^(^ ^34^ ^)^ Because testosterone in the absence of estrogen was shown to stimulate osteoblast apoptosis,^(^ ^35^ ^)^ the hormonal imbalance induced by AIs could be hypothesized as the mechanism of increased bone fragility in women with high‐LBM and low‐FBM in this study.^(^ ^36^ ^)^ Unfortunately, sex hormone levels were not measured in our study cohort, so this hypothesis deserves to be further explored.

### Limitations

The cross‐sectional design is the major limitation of the present study because we cannot assess whether VFs occurred before or during AIs therapy in the AI group. Similarly, it is not possible to demonstrate if changes in the body composition appeared before or after the VF.

Although the number of patients recruited in this single‐institution study is relevant, and the introduction of the propensity score adjusted for differences between groups, our findings should be considered exploratory and deserve confirmation in a longitudinal prospective study.

Furthermore, the LBM measurement carried out with DXA is indirect, depending on the X‐rays' attenuation and may be influenced by the tissue thickness with a potential overestimation of muscle mass in obese patients.^(^ ^37^ ^)^

## Conclusions

The present observational study provides additional data supporting the notion that bone fragility in women under AI therapy has different pathophysiology than postmenopausal osteoporosis. Our data suggest that identifying the body composition phenotype with low‐LBM and high‐FBM could significantly improve the prediction and management of fracture risk in AI‐induced osteopathy.

## Disclosures

The Authors declare no conflicts of interest.

## AUTHOR CONTRIBUTIONS

**Sara Monteverdi:** Conceptualization; data curation; formal analysis; writing‐original draft; writing‐review and editing. **Rebecca Pedersini:** Conceptualization; data curation; formal analysis; writing‐original draft; writing‐review and editing. **Fabio Gallo:** Formal analysis; writing‐review and editing. **Filippo Maffezzoni:** Data curation; investigation; writing‐review and editing. **Alberto Dalla Volta:** Writing‐review and editing. **Pierluigi Di Mauro:** Writing‐review and editing. **Antonella Turla:** Data curation; writing‐review and editing. **Lucia Vassalli:** Data curation; writing‐review and editing. **Mara Ardine:** Investigation; writing‐review and editing. **Anna Maria Formenti:** Conceptualization; writing‐review and editing. **Edda Lucia Simoncini:** Conceptualization; funding acquisition; supervision. **Andrea Giustina:** Conceptualization; methodology; writing‐review and editing. **Roberto Maroldi:** Conceptualization; supervision; writing‐review and editing. **Vito Amoroso:** Conceptualization; formal analysis; methodology; supervision; writing‐original draft; writing‐review and editing. **Alfredo Berruti:** Conceptualization; formal analysis; funding acquisition; methodology; supervision; writing‐original draft; writing‐review and editing.

### Peer Review

The peer review history for this article is available at https://publons.com/publon/10.1002/jbm4.10440.

## Supporting information


**Table S1.** Demographic and Clinical Characteristics of AI‐naïve and AI‐treated Breast Cancer Patients Before (*n* = 684) and After Propensity Score Nearest Neighbour Matching (*n* = 480).
**Table S2.** Contingency Tables and Output of the Univariate Analysis After Propensity Score Nearest Neighbour Matching (*n* = 480).
**Fig. S3.** Prevalence odds ratios with 95% confidence interval of the chance of a prevalent vertebral fracture among AI‐naïve and AI‐treated patients with covariate adjustment in the propensity score‐matched sample (*n* = 480).
**Table S4.** Multivariable Logistic Regression Model With Lean Body Mass and Fat Body Mass Treated as Continuous Variables in the Propensity Score‐Matched Sample.
**Fig. S5.** Predicted probability of vertebral fracture based on lean body mass as a continuous variable with fat body mass fixed at the highest or lowest value in the AI‐naïve and AI‐treated group (A and B, respectively).
