# Supplementary material for: The Interaction of Lean Body Mass With Fat Body Mass Is Associated With Vertebral Fracture Prevalence in Women With Early Breast Cancer Undergoing Aromatase Inhibitor Therapy
Source: JBMR Plus. 2020 Dec 21;5(2):e10440. doi: 10.1002/jbm4.10440 (PMC7872339; doi:10.1002/jbm4.10440)
Supplement: Supplementary file 1 — Table S1. Demographic and Clinical Characteristics of AI‐naïve and AI‐treated Breast Cancer Patients Before (n = 684) and After Propensity Score Nearest Neighbour Matching (n = 480). Table S2. Contingency Tables and Output of the Univariate Analysis After Propensity Score Nearest Neighbour Matching (n = 480). Fig. S3. Prevalence odds ratios with 95% confidence interval of the chance of a prevalent vertebral fracture among AI‐naïve and AI‐treated patients with covariate adjustment in the propensity score‐matched sample (n = 480). Table S4. Multivariable Logistic Regression Model With Lean Body Mass and Fat Body Mass Treated as Continuous Variables in the Propensity Score‐Matched Sample. Fig. S5. Predicted probability of vertebral fracture based on lean body mass as a continuous variable with fat body mass fixed at the highest or lowest value in the AI‐naïve and AI‐treated group (A and B, respectively). [file JBM4-5-e10440-s001.docx]

**Supplementary materials**

**Table S1**. Demographic and Clinical Characteristics of AI-naïve and AI-treated Breast Cancer Patients before (n = 684) and after Propensity Score nearest Neighbour Matching (n = 480).

**Table S2**. Contingency Tables and Output of the Univariate Analysis after Propensity Score nearest Neighbour Matching (n = 480).

**Figure S3**. Prevalence Odds Ratios with 95% Confidence Interval of the Chance of a Prevalent Vertebral Fracture among AI-naïve and AI-treated Patients with Covariate Adjustment in the Propensity score-matched Sample (n = 480).

**Table S4**. Multivariable Logistic Regression Model with Lean Body Mass and Fat Body Mass treated as Continuous Variables in the Propensity score-matched Sample.

**Figure S5**. Predicted Probability of Vertebral Fracture based on Lean Body Mass as a Continuous Variable with Fat Body Mass fixed at the Highest or Lowest Value in the AI-naïve and AI-treated Group (panel A and B, respectively).

**Table S1**. Demographic and Clinical Characteristics of AI-naïve and AI-treated Breast Cancer Patients before (n = 684) and after Propensity Score nearest Neighbour Matching (n = 480).

|  | **Before matching** | | | | **After matching** | |
| --- | --- | --- | --- | --- | --- | --- |
| **Characteristic** | **Descriptive statistic** | | **Univariate analysis** | | **Univariate analysis** | |
|  | **AI group** | | **OR (95% CI)** | ****P*-value** | **OR (95% CI)** | ****P*-value** |
|  | *AI-naïve*  *439 (64.2%)* | *AI-treated*  *245 (35.8%)* |  |  |  |  |
| Morphometric  vertebral fracture |  |  |  | .019 |  | .99 |
| *Absence* | 370 (84.3%) | 181 (73.9%) | 1 |  | 1 |  |
| *Presence* | 69 (15.7%) | 64 (26.1%) | 1.9 (1.29 - 2.78) |  | 1.49 (0.97 - 2.3) |  |
| Vertebral fracture grade |  |  |  | .07 |  | .99 |
| *No fracture* | 370 (84.3%) | 181 (73.9%) | 1 |  | 1 |  |
| *Mild fractures* | 33 (7.5%) | 33 (13.5%) | 2.04 (1.22 - 3.43) |  | 1.64 (0.93 - 2.96) |  |
| *Moderate/severe fractures* | 36 (8.2%) | 31 (12.6%) | 1.76 (1.05 - 2.94) |  | 1.36 (0.77 - 2.41) |  |
| No. of vertebral fractures |  |  |  | .006 |  | .91 |
| *No fracture* | 370 (84.3%) | 181 (73.9%) | 1 |  | 1 |  |
| *One fracture* | 51 (11.6%) | 35 (14.3%) | 1.4 (0.88 - 2.23) |  | 1.16 (0.69 - 1.96) |  |
| *Two or more fractures* | 18 (4.1%) | 29 (11.8%) | 3.29 (1.8 - 6.19) |  | 2.27 (1.18 - 4.56) |  |
| Physical activity |  |  |  | .011 |  | .99 |
| *No* | 295 (78.0%) | 159 (65.4%) | 1 |  | 1 |  |
| *Yes* | 83 (22.0%) | 84 (34.6%) | 1.88 (1.31 - 2.69) |  | 1.18 (0.81 - 1.74) |  |
| Smoking status |  |  |  | .99 |  | .99 |
| *No* | 352 (84.0%) | 207 (84.8%) | 1 |  | 1 |  |
| *Yes* | 67 (16.0%) | 37 (15.2%) | 0.94 (0.6 - 1.45) |  | 0.84 (0.51 - 1.35) |  |
| Alcohol consumption |  |  |  | <.001 |  | .81 |
| *No* | 331 (83.4%) | 169 (69.5%) | 1 |  | 1 |  |
| *Yes* | 66 (16.6%) | 74 (30.4%) | 2.2 (1.5 - 3.22) |  | 1.54 (1.02 - 2.32) |  |
| **Pathologic tumor stage** |  |  |  | .99 |  | .99 |
| *pT1* | 290 (66.8%) | 177 (72.8%) | 1 |  | 1 |  |
| *pT2* | 128 (29.5%) | 58 (23.9%) | 0.74 (0.51 - 1.06) |  | 0.71 (0.47 - 1.06) |  |
| *pT3-4* | 16 (3.7%) | 8 (3.3%) | 0.82 (0.33 - 1.9) |  | 1.84 (0.57 - 7) |  |
| **Pathologic nodal status** |  |  |  | .99 |  | .99 |
| *pN0* | 256 (59.5%) | 149 (61.8%) | 1 |  | 1 |  |
| *pN1* | 153 (35.6%) | 72 (29.9%) | 0.81 (0.57 - 1.14) |  | 0.97 (0.65 - 1.44) |  |
| *pN2-3* | 21 (4.9%) | 20 (8.3%) | 1.64 (0.85 - 3.13) |  | 1.44 (0.7 - 3.01) |  |
| Chemotherapy use |  |  |  | <.001 |  | .99 |
| *No* | 244 (55.6%) | 178 (73.2%) | 1 |  | 1 |  |
| *Yes* | 195 (44.4%) | 65 (26.7%) | 0.46 (0.32 - 0.64) |  | 0.83 (0.56 - 1.24) |  |
| Previous **Fractures** |  |  |  | .003 |  | .99 |
| *No* | 363 (86.2%) | 180 (74.4%) | 1 |  | 1 |  |
| *Yes* | 58 (13.8%) | 62 (25.6%) | 2.16 (1.45 - 3.22) |  | 1.39 (0.91 - 2.15) |  |
| **Age** | 61.19 (10.5) | 66.27 (7.86) | 1.06 (1.04 - 1.08) | <.001 | 1.01 (0.99 - 1.04) | .99 |
| **BMI** | 25.29 (4.71) | 26.34 (4.48) | 1.05 (1.01 - 1.09) | .07 | 1.01 (0.97 - 1.05) | .99 |
| Lumbar spine BMD, (g/cm^2^ ) | 0.9 (0.15) | 0.88 (0.12) | 0.33 (0.11 - 1.01) | .61 | 0.39 (0.11 - 1.38) | .99 |
| Lumbar spine T-score | -1.22 (1.48) | -1.51 (1.1) | 0.85 (0.76 - 0.96) | .11 | 0.85 (0.74 - 0.97) | .30 |
| Femoral neck BMD, *(g/cm^2^)* | 0.69 (0.11) | 0.68 (0.09) | 0.25 (0.05 - 1.2) | .93 | 0.3 (0.05 - 1.84) | .99 |
| Femoral neck T-score | -1.39 (0.96) | -1.5 (0.79) | 0.88 (0.74 - 1.05) | .99 | 0.9 (0.73 - 1.1) | .99 |
| Total Hip BMD, *(g/cm^2^)* | 0.82 (0.11) | 0.81 (0.1) | 0.51 (0.12 - 2.18) | .99 | 0.42 (0.08 - 2.33) | .99 |
| Total Hip T-score | -0.98 (0.95) | -1.07 (0.78) | 0.89 (0.75 - 1.07) | .99 | 0.87 (0.71 - 1.07) | .99 |
| Lean Body Mass, *(grams)* | 39858.91 (5172.4) | 40189.53 (4861.4) | 1 (0.99 - 1.01) | .99 | 1 (0.99 - 1.01) | .99 |
| Fat Body Mass, *(grams)* | 25877.96 (28783.7) | 26674.95 (8713.5) | 1 (0.99 - 1.01) | .99 | 1 (0.99 - 1.01) | .99 |

*Likelihood ratio *P*-value adjusted for multiple comparisons.

Abbreviations: AI, aromatase inhibitor; BMD, bone mineral density; BMI, body mass index.

**Table S2**. Contingency Tables and Output of the Univariate Analysis after Propensity Score nearest Neighbour Matching (n = 480).

| **Characteristic** | **Descriptive statistic** | | **Univariate analysis** | |
| --- | --- | --- | --- | --- |
|  | Morphometric vertebral fracture | | **OR (95% CI)** | *****P*-value** |
|  | *Absence*  *369 (76.9%)* | *Presence*  *111 (23.1%)* |  |  |
| **AI group** |  |  |  | .99 |
| *AI-naïve* | 193 (52.3%) | 47 (42.3%) | 1 |  |
| *AI-treated* | 176 (47.7%) | 64 (57.7%) | 1.49 (0.97 - 2.3) |  |
| Physical activity |  |  |  | .99 |
| *No* | 245 (66.4%) | 76 (68.5%) | 1 |  |
| *Yes* | 124 (33.6%) | 35 (31.5%) | 0.91 (0.57 - 1.43) |  |
| Smoking status |  |  |  | .99 |
| *No* | 305 (82.7%) | 95 (85.6%) | 1 |  |
| *Yes* | 64 (17.3%) | 16 (14.4%) | 0.8 (0.43 - 1.42) |  |
| Alcohol consumption |  |  |  | .99 |
| *No* | 275 (74.5%) | 77 (69.4%) | 1 |  |
| *Yes* | 94 (25.5%) | 34 (30.6%) | 1.29 (0.8 - 2.05) |  |
| **Pathologic tumor stage** |  |  |  | .99 |
| *pT1* | 251 (68.2%) | 87 (78.4%) | 1 |  |
| *pT2* | 106 (28.8%) | 23 (20.7%) | 0.63 (0.37 - 1.03) |  |
| *pT3-4* | 11 (3.0%) | 1 (0.9%) | 0.26 (0.01 - 1.38) |  |
| **Pathologic nodal status** |  |  |  | .99 |
| *pN0* | 225 (61.6%) | 72 (66.1%) | 1 |  |
| *pN1* | 109 (29.7%) | 34 (31.2%) | 0.97 (0.61 - 1.55) |  |
| *pN2-3* | 31 (8.5%) | 3 (2.7%) | 0.3 (0.07 - 0.88) |  |
| Chemotherapy use |  |  |  | .22 |
| *No* | 255 (69.1%) | 90 (81.1%) | 1 |  |
| *Yes* | 114 (30.9%) | 21 (18.9%) | 0.52 (0.3 - 0.87) |  |
| Previous **fractures *** |  |  |  | <.001 |
| *No* | 306 (82.9%) | 64 (57.7%) | 1 |  |
| *Yes* | 63 (17.1%) | 47 (42.3%) | 3.57 (2.24 - 5.68) |  |
| **Age *** | 65.1 (7.6) | 69.5 (7.2) | 1.08 (1.05 - 1.12) | <.001 |
| **BMI** | 26.4 (4.6) | 26.3 (4.7) | 1 (0.95 - 1.04) | .99 |
| Lumbar spine BMD, (g/cm^2^) | 0.89 (0.14) | 0.89 (0.16) | 0.88 (0.19 - 3.83) | .99 |
| Lumbar spine T-score | -1.37 (1.29) | -1.31 (1.61) | 1.03 (0.88 - 1.2) | .99 |
| Femoral neck BMD, *(g/cm^2^)* | 0.69 (0.1) | 0.67 (0.11) | 0.1 (0.01 - 0.86) | .61 |
| Femoral neck T-score | -1.41 (0.85) | -1.62 (0.98) | 0.76 (0.59 - 0.97) | .49 |
| Total Hip BMD, *(g/cm^2^) ** | 0.83 (0.1) | 0.79 (0.11) | 0.03 (0.01 - 0.23) | .016 |
| Total Hip T-score | -0.95 (0.85) | -1.26 (0.9) | 0.64 (0.49 - 0.83) | .015 |
| Lean Body Mass, *(grams)* | 40376.1 (5036.1) | 40091.86 (5333.9) | 1 (0.99 - 1.01) | .99 |
| Fat Body Mass, *(grams)* | 26497.89 (8351.7) | 26488.25 (9051.8) | 1 (0.99 - 1.01) | .99 |
| **Body Mass** |  |  |  | .99 |
| *Group A, LBM- & FBM-* | 136 (36.9%) | 42 (37.8%) | 1 |  |
| *Group B, LBM- & FBM+* | 42 (11.4%) | 20 (18.0%) | 1.54 (0.81 - 2.89) |  |
| *Group C, LBM+ & FBM-* | 46 (12.5%) | 15 (13.5%) | 1.06 (0.52 - 2.05) |  |
| *Group D, LBM+ & FBM+* | 145 (39.3%) | 34 (30.6%) | 0.76 (0.45 - 1.26) |  |

*Variables entering the multivariable analysis (see the text for further details). **Likelihood ratio *P*-value.

Abbreviations: AI, aromatase inhibitor; BMD, bone mineral density; BMI, body mass index; LMB-, lean body mass < median value; LBM+, lean body mass ≥ median value; FBM- fat body mass < median value; FBM+, fat body mass ≥ median value.

**Figure S3**. Prevalence Odds Ratios with 95% Confidence Interval of the Chance of a Prevalent Vertebral Fracture among AI-naïve and AI-treated Patients with Covariate Adjustment in the Propensity score-matched Sample (n = 480).


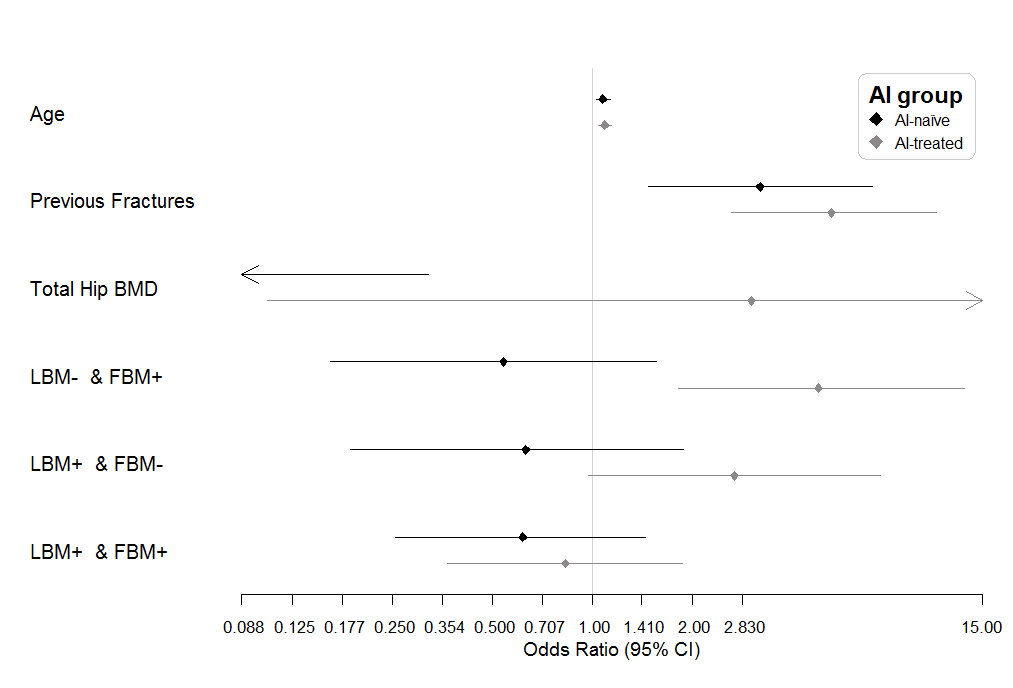


Abbreviations: AI, aromatase inhibitor; BMD, bone mineral density; CI, confidence interval; LMB-, lean body mass < median value; LBM+, lean body mass ≥ the median value; FBM-, fat body mass < median value; FBM+, fat body mass ≥ median value.

**Table S4**. Multivariable Logistic Regression Model with Lean Body Mass and Fat Body Mass treated as Continuous Variables in the Propensity score-matched Sample.

| **Characteristic** | **Multivariable model** | |
| --- | --- | --- |
|  | **OR (95% CI)** | ****P*-value** |
| **Age** | 1.08 (1.05 - 1.12) | <.0001 |
| Previous **fractures** |  | <.0001 |
| *No* | 1 |  |
| *Yes* | 3.9 (2.33 - 6.56) |  |
| Total hip BMD, *(g/cm^2^)* | 0.17 (0.01 - 2.25) | .0321 |
| **AI group** |  | .1821 |
| *AI-naïve* | 1 |  |
| *AI-treated* | 0 (0 - 1.03) |  |
| Log(FBM) | 0 (0 - 241525.04) | .8803 |
| Log(LBM) | 0 (0 - 344241.51) | .3584 |
| **AI group** * Log(FBM) |  | .0283 |
| *AI-naïve /* Log(*FBM)* | 1 |  |
| *AI-treated /* Log(*FBM)* | 5.02e+20 (542.9 - 1.02e+40) |  |
| **AI group *** Log(**LBM)** |  | .2757 |
| *AI-naïve /* Log(*LBM)* | 1 |  |
| *AI-treated /* Log(*LBM)* | 1.77 (1.64 - 1.88e+33) |  |
| Log(LBM) * Log(FBM) | 19.78 (0.03 - 19614.34) | .5324 |
| **AI group** * Log(LBM) * Log(FBM) |  | .0311 |
| *AI-naïve /* Log(*LBM) /* Log(*FBM)* | 1 |  |
| *AI-treated /* Log(*LBM) /* Log(*FBM)* | 0.01 (0 - 0.34) |  |

Results are expressed as odds ratios (OR) with 95% confidence interval (95% CI). *Likelihood ratio *P*-value.

Abbreviations: AI, aromatase inhibitor; BMD, bone mineral density; LMB, lean body mass; FBM, fat body mass.

**Figure S5**. Predicted Probability of Vertebral Fracture based on Lean Body Mass as a Continuous Variable with Fat Body Mass fixed at the Highest or Lowest Value in the AI-naïve and AI-treated Group (panel A and B, respectively).

| **A:** **AI-naïve** | **B: AI-treated** |
| --- | --- |
| 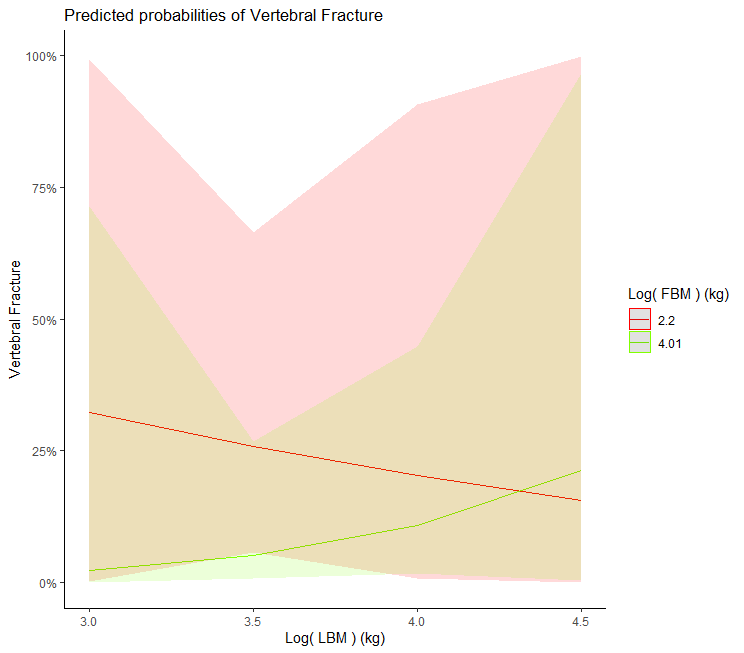 | 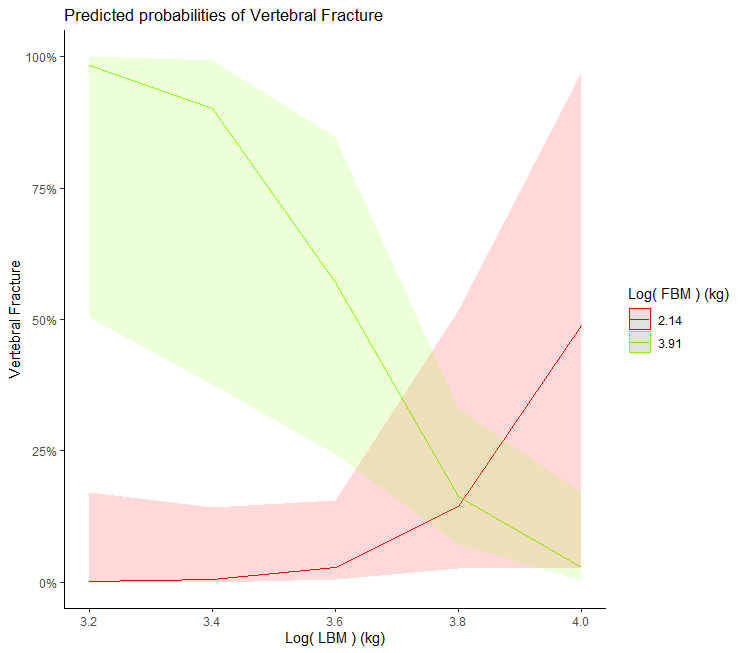 |
